# Recent advances in gradient biomimetic scaffolds for tendon-bone interface regeneration

**DOI:** 10.3389/fbioe.2025.1629816

**Published:** 2025-09-09

**Authors:** Xianyan Xie, Yu Wang, Ziyan Li, Gaoyuan Yang, Guodong Cheng, Shuqi Qin, Huiguo Wang, Lin Zhu

**Affiliations:** ^1^ College of Sport and Health, GuangZhou Sport University, Guangzhou, China; ^2^ Research Center for Innovative Development of Sports and Healthcare Integration, GuangZhou Sport University, Guangzhou, China; ^3^ Innovative Research Center for Sports Science in the Guangdong-Hong Kong-Macao Greater Bay Area, GuangZhou Sport University, Guangzhou, China

**Keywords:** tendon-bone interface regeneration, gradient biomimetic scaffold, tissue engineering, advanced fabrication techniques, translational regenerative medicine

## Abstract

Injury and repair of the tendon-bone interface (TBI) pose a significant challenge in the fields of orthopedics and sports medicine. Due to the gradients in structure, composition, mechanical properties, and biological signals across the TBI, transitioning from flexible tendon to rigid bone, traditional surgical approaches often struggle to reconstruct its functional structure, leading to poor mechanical properties of the interface after repair and high re-tear rates. In contrast, gradient biomimetic scaffolds, by mimicking the continuous gradients of native TBI, offer an effective solution for achieving functional TBI regeneration. This review systematically summarizes the research progress of gradient biomimetic scaffolds for TBI regeneration in recent years. Firstly, we discuss the fine structure, physiological functions of native TBI, and the repair challenges faced after its injury, emphasizing the necessity of reconstructing gradient structures. Subsequently, it focuses on the design principles and core biomimetic strategies of gradient biomimetic scaffolds, discussing in depth the principles of construction and implementation strategies for physical structure gradients (e.g., porosity, fiber orientation, mechanical modulus), chemical composition gradients (e.g., degree of mineralization, polymer/ECM components), and biological signal gradients (e.g., growth factors, genes). Building upon this, this review comprehensively reviews various biomaterials for gradient scaffold construction, including natural polymers (collagen, silk fibroin, chitosan, etc.), synthetic polymers (PCL, PLGA, PU, etc.), and inorganic bioactive materials (calcium phosphate ceramics, bioactive glass), analyzing their characteristics, functionalization methods, and applications in gradient construction. Furthermore, this review also systematically summarizes and compares major fabrication techniques for gradient biomimetic scaffolds, particularly the advantages and limitations of electrospinning and additive manufacturing (3D printing) in constructing specific gradient features, and highlights emerging techniques such as microfluidics. Finally, building upon the summarized existing research findings, this review critically analyzes the key challenges and technical bottlenecks currently facing gradient biomimetic scaffolds regarding structural biomimetic accuracy, spatio-temporal control of biological functions, vascularization, and immunocompatibility, and offers perspectives on future research directions, such as smart scaffolds, integration of multiple gradients, personalized manufacturing, and clinical translation strategies.

## 1 Introduction

The tendon-bone interface (TBI), a specialized region where tendons or ligaments attach to bone, is primarily responsible for effectively transmitting mechanical forces generated by muscles (Killian, 2022). This interface possesses a unique, layered and graded structure transitioning from flexible tendon to rigid bone, accompanied by continuous gradient variations in cell types, extracellular matrix components, and degree of mineralization (Dang et al., 2022). However, under conditions of chronic overload or acute trauma (such as sports injuries), the intricate gradient structure of the TBI is highly susceptible to damage, leading to interface injury (Deng et al., 2019; Lyons et al., 2024).

Tendon-bone interface injuries are common and prevalent within the musculoskeletal system, causing significant impact on patients’ quality of life and athletic ability. Latest epidemiological data show that these types of injuries have a high incidence. For instance, rotator cuff tears alone affect millions of people globally each year, with a prevalence of full-thickness tears reaching 22.2% in women aged 64–87 (Hinsley et al., 2022), and their incidence significantly increases with age (Xu F. et al., 2024; Fehringer et al., 2008). Similarly, anterior cruciate ligament injuries are also extremely prevalent, particularly among individuals participating in sports activities (Goenka et al., 2019). In the United States, the incidence of ACL tears is approximately 68.6 per 100,000 people annually (Sanders et al., 2016), with approximately 129,000 ACL reconstruction surgeries performed each year (Gavia et al., 2023). These injuries not only lead to significant pain and functional disability but also impose a substantial socioeconomic burden, including high medical costs, rehabilitation expenses, and lost productivity due to missed work (Adjei-Sowah et al., 2023).

Currently, the primary method for repairing severe tendon-bone interface injuries clinically is surgical intervention, aiming to reattach the torn tendon or ligament to the bone (Yang et al., 2023). However, traditional surgical methods face significant challenges, and their clinical outcomes are often unsatisfactory. The main limitations include: ① High post-operative re-tear rates (Heuberer et al., 2017) [e.g., reported re-tear rates after rotator cuff repair can range from 20% to 94% (Zhao et al., 2017)]; ② The repaired interface tends to form mechanically inferior fibrotic scar tissue, composed of disorganized collagen fibres. This results in an ultimate tensile strength and elastic modulus significantly lower than that of native tissue (Bai et al., 2022). Moreover, this tissue fails to recapitulate the graded transition from soft tissue to hard bone, leading to the regeneration of a non-functional tendon-bone insertion (Huang et al., 2021); ③ and due to structural mismatch, stress concentration occurs at the repair site, increasing the risk of re-injury (Avgoulas et al., 2019). The root cause of these issues lies in the difficulty of existing techniques in reconstructing the complex gradient structure and biological microenvironment unique to native TBI.

To address these challenges, researchers have explored various adjuvant and alternative treatment strategies. However, these approaches generally lack precise control over biological signalling and struggle to fully replicate and reconstruct the complex multi-scale gradient structure and functional integration of the TBI. Therefore, there is a need for more comprehensive and biomimetic solutions.

In recent years, with the rapid development of the fields of tissue engineering, biomaterials science, and biomimetics, gradient biomimetic scaffolds (Gradient Biomimetic Scaffolds), as an advanced tissue engineering strategy, have become a research focus in the field of TBI repair (Fang et al., 2024; Liu et al., 2025). The core concept of gradient biomimetic scaffolds lies in mimicking the continuous changes in structure, composition, mechanical properties, and biological signaling molecules from the tendon to the bone end of native TBI (Liu et al., 2025). By providing a microenvironment with spatially specific cues, these scaffolds aim to guide the ordered behavior of host cells or transplanted cells (such as proliferation, migration, and directed differentiation), promote vascularization, and ultimately achieve TBI regeneration with a functional gradient transition zone (Bai et al., 2022; Kim et al., 2025; Longfei et al., 2022). Compared to conventional homogeneous scaffolds, gradient biomimetic scaffolds, by more accurately mimicking the structural features and biological functions of native tissue, are expected to more effectively promote cell lineage-specific differentiation, ordered tissue regeneration, and restore the interface’s mechanical load transfer capacity, demonstrating significant clinical application potential (Xiping Jiang et al., 2022; Yea et al., 2020).

This review aims to systematically review the latest research progress on gradient biomimetic scaffolds for tendon-bone interface regeneration. We will focus on the following four aspects (Killian, 2022): The tissue structural characteristics, physiological functions, and key scientific challenges faced in injury repair of the tendon-bone interface (Dang et al., 2022); The design principles and core biomimetic construction strategies (physical, chemical, biological signal gradients) of gradient biomimetic scaffolds (Deng et al., 2019); Various biomaterial systems suitable for gradient scaffold construction and their functionalization and modification methods; and (Lyons et al., 2024) Major fabrication techniques for gradient biomimetic scaffolds and their advantages and disadvantages. By deeply analyzing existing research findings, this paper will analyze the key scientific problems and technical bottlenecks that need to be addressed in this field, and provide a forward-looking outlook on future research directions, in order to provide valuable reference for promoting the development of tendon-bone interface regenerative medicine.

## 2 Structure, function, and challenges in injury repair of the tendon-bone interface

The tendon-bone interface (TBI), also known as the enthesis, is a unique biological composite structure connecting tendons or ligaments to bone (Killian, 2022). Its core function lies in effectively transmitting mechanical forces generated by muscles to the skeletal system, while simultaneously adapting to different types of mechanical loads (Killian, 2022). A deep understanding of the TBI’s tissue structural characteristics, unique mechanical load transfer mechanisms, and the pathophysiological processes after injury is fundamental to developing effective regeneration and repair strategies.

### 2.1 Gradient structural characteristics and stress transfer mechanisms of the tendon-bone interface

**FIGURE 1:**
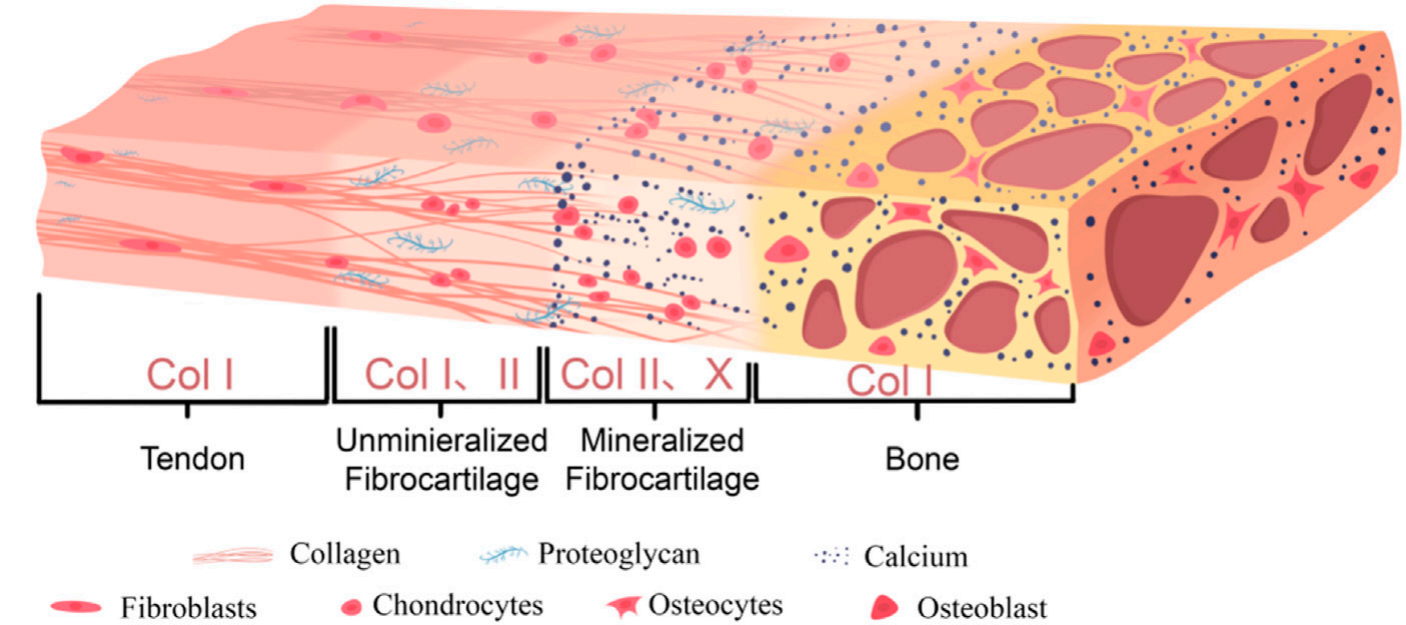
Graded structure of the tendon-bone interface.

A typical tendon-bone interface is usually composed of four continuous and progressively transitioning zones: tendon, unmineralized fibrocartilage, mineralized fibrocartilage, and bone tissue (Tits et al., 2023). Figure 1 shows the graded structure of the tendon-bone interface and Table 1 shows the microstructural arrangement and mechanical properties within each layer of the tendon-bone interface and their relationship with injury.

**TABLE 1:** The microstructural arrangement and mechanical properties within each layer of the tendon-bone interface and their relationship with injury.

Layer	Microstructure			Mechanical properties			Relationship with injury/Degeneration
	Cell type	ECM components	Structural features	Elastic modulus	Stress characteristics	Main function	
Tendon Region	Tenocytes	Type I Collagen, Decorin	Collagen fibres are highly parallel and densely arranged	200–400 MPa	High tensile strength, weak compressive/shear strength	Efficiently transmits tensile stress generated by muscles	Excessive tensile loading or rapid impact force can cause micro-tears or macroscopic rupture of tendon fibres
Uncalcified Fibrocartilage Region	Round chondrocyte-like cells	Type I Collagen, Type II Collagen, Aggrecan	Collagen fibres begin to become disordered and interwoven, with uniform angular deviation	Gradually increasing, but remains flexible	Begins to exhibit resistance to compression and shear	Acts as a stress “cushion”, dispersing unidirectional tensile stress into multidirectional complex stress	Prone to matrix degradation and microstructural tearing under complex stress, leading to reduced compressive buffering capacity
Calcified Fibrocartilage Region	Flattened chondrocyte-like cells	Type II Collagen, Type X Collagen, Type I Collagen, Proteoglycans, Hydroxyapatite	Collagen fibres are mineralized; “tidemark” appears	Sharply increasing modulus, significant increase in hardness	Strong resistance to compressive and shear stress	Enables a smooth transition in mechanical properties from flexible tissue to rigid tissue	Mineralization abnormalities or degenerative calcification cause increased brittleness at the interface, leading to delamination or avulsion under shear stress Blurring of the tidemark indicates scar tissue formation and reduced interface repair capacity
Bone Region	OsteoblastsOsteocytes	Type I Collagen highly mineralized matrix	Collagen fibres are fully mineralized, forming woven or lamellar bone; Sharpey’s fibres appear	Up to 20 GPa	Provides ultimate rigid fixation and support	Distributes load stably throughout the skeletal system	Osteoporosis weakens bone quality, reducing anchoring strength Poor bone tunnel healing is a cause of graft fixation failure

The tendon zone is primarily composed of highly parallel, dense bundles of type I collagen fibers, with cell components mainly consisting of longitudinally aligned tenocytes. The extracellular matrix (ECM) is rich in type I collagen and contains a small amount of proteoglycans such as Decorin (Tu et al., 2020; Giacomini et al., 2024). This zone primarily bears tensile stress and exhibits excellent tensile properties (Wang H. et al., 2024).

Transitioning into the unmineralized fibrocartilage zone, fiber angle deviation shows relative uniformity throughout the attachment site region, although certain regional differences exist (e.g., distinct tissue organization between the central and peripheral areas) (Thomopoulos et al., 2006). Cell morphology changes to oval-shaped chondrocyte-like cells (Pitta et al., 2023). In the ECM, in addition to type I collagen, type II collagen, which is more resistant to compressive stress, begins to appear, along with a small amount of proteoglycans (such as Aggrecan), but mineralization has not yet occurred (Zhu et al., 2021). During physiological activity, tendons do not experience purely tensile forces. When a tendon is tensioned and wraps around a bony prominence (forming an anatomical pulley system, such as the Achilles tendon around the calcaneus), its deep layers are subjected to significant compressive stress (Bol et al., 2024). Furthermore, during complex joint movements involving bending, twisting, or turning, the tendon fascicles undergo relative sliding or experience torsional loading, resulting in substantial shear stress (Reiter et al., 2024). In summary, these compositional and structural changes enable this region to withstand and buffer compressive and shear stresses (Pitta et al., 2023).

In the mineralized fibrocartilage zone, fibrochondrocytes are embedded in significantly mineralized ECM (Pitta et al., 2023), with relatively lower cell density and activity. Type II and type X collagen are characteristic molecular markers for this region, which also contains some type I collagen and proteoglycans (Pitta et al., 2023). A substantial deposition of minerals (primarily hydroxyapatite) in this region forms a clearly defined “tidemark.” This structure not only serves as a physical demarcation between the uncalcified and calcified zones (Pitta et al., 2023), but also acts as a functional barrier separating the avascular microenvironment from the vascular-rich microenvironment (Yildirim et al., 2023). The stiffness of this region significantly increases, further adapting to stress and promoting integration with the underlying bone tissue (Tits et al., 2023; Pitta et al., 2023).

Finally transitioning to the bone zone, the mineralized fibrocartilage tightly integrates with the underlying cortical or cancellous bone (Tits et al., 2021). This integration is facilitated by specialized bone morphology at the attachment site (such as distinct bony prominences) and microstructural anisotropy exhibited within both the mineralized fibrocartilage and subchondral bone, including specific arrangements of lacunae and bone channels (Tits et al., 2021). Sharpey’s fibers in this region penetrate deep into the bone tissue, facilitating the establishment of a stronger tendon-bone connection, thereby improving force transmission (Jiang et al., 2023). Furthermore, osteoblasts and osteocytes in this zone are embedded in mineralized type I collagen and minerals (Zhu et al., 2021).

This continuous gradient transition from flexible tendon to rigid bone (including gradients in cell phenotype, ECM composition, degree of mineralization, and mechanical properties) is the key structural basis that enables the TBI to effectively dissipate interface stress, avoid stress concentration, and achieve efficient mechanical load transfer.

### 2.2 Pathophysiology and natural healing challenges of tendon-bone interface injuries

Tendon-bone injuries are common conditions within the musculoskeletal system, which can result from acute high-intensity trauma (e.g., sudden tendon tears during sports) or chronic cumulative overuse (e.g., enthesopathy caused by degenerative tendinopathy) (Deng et al., 2019; Lyons et al., 2024). Common types of TBI injuries include rotator cuff tears (Kaizawa et al., 2019), Achilles enthesopathy (Zhang et al., 2019), and anterior cruciate ligament (ACL) avulsion (Hexter et al., 2020). Compared to other relatively well-vascularized soft tissues, the TBI exhibits extremely limited natural healing capacity (Wang H. et al., 2024). Its healing impairment is primarily attributed to several interconnected factors:

Poor Blood Supply: The fibrocartilage zone is generally considered an avascular structure or one with only a very limited microvascular distribution (Shea et al., 2002), which severely restricts the effective delivery of nutrients, growth factors, and repair cells (such as immune cells, stem/progenitor cells) to the injury site.

Dysfunctional scarring and structural disorganization: Following TBI injury, the body’s natural repair response often fails to guide the regeneration of functional tissue, instead tending to form mechanically inferior fibrous scar tissue (Zhang et al., 2023a; Smatov et al., 2023; Adeoye et al., 2022). This scar tissue is primarily composed of disorganized Type I and III collagen fibers (Zhang J. et al., 2023), resulting in a failure to replicate the multi-zone layered structure and molecular gradients unique to native TBI, particularly lacking the crucial fibrocartilage transition zone and mineralization gradient. Tendons experience complex cyclic loading during daily activities. For example, the human Achilles tendon can be subjected to peak forces of approximately 44.0 N/kg during walking and 98.7 N/kg during running (Reiter et al., 2024). The scar tissue interface formed after repair exhibits reduced ultimate tensile strength and is prone to plastic deformation and even failure at lower strains (Dai et al., 2024). This leads to a repaired interface with mechanical strength significantly lower than that of native tissue, rendering it highly susceptible to re-tear under physiological loading (Sun et al., 2020).

Disruption of the biological microenvironment: Dysregulated inflammatory responses (e.g., persistent, non-resolving inflammation) (Gao et al., 2023), insufficient recruitment of endogenous stem/progenitor cells or impaired directed differentiation towards the tendon-bone lineage (Zuo et al., 2022), and unfavorable mechanical microenvironment post-injury (e.g., stress shielding or abnormal stress distribution) (Wang C. et al., 2024) can all further impede the regeneration of functional TBI.

Disrupted Nerve Regeneration and Dysregulation: A rich network of nerve endings forms a sophisticated sensory and regulatory network within the native tendon-bone interface (Xu M. et al., 2024). Following tendon-bone injury, this network is severely disrupted, resulting in severed nerve fibres, disordered nerve regeneration, and dysregulation of neural signalling (Zhao X. et al., 2024; Li et al., 2024). This disruption can lead to a deficiency in pro-regenerative signals, chronic pain, and incomplete functional recovery, ultimately impacting tendon-bone healing.

### 2.3 *In vivo* evaluation of tendon-bone interface repair using animal models

Animal models play a crucial role in translating biomimetic scaffolds from laboratory design to clinical application. Commonly used animal models for TBI repair include small animal models (e.g., rat rotator cuff injury, rabbit anterior cruciate ligament reconstruction) and large animal models (e.g., sheep rotator cuff injury). These models can simulate the biological responses, mechanical loading, and tissue healing processes that occur after tendon-bone interface injury in the complex *in vivo* environment, providing direct evidence for evaluating the efficacy and biocompatibility of biomimetic scaffolds. A summary of the effectiveness and biocompatibility of tendon-bone injury repair based on animal models is presented in Table 2.

**TABLE 2:** Efficacy and biocompatibility of graded scaffolds for tendon-bone interface in animal models.

Animal	Model	Advantages	Disadvantages	Suitability for Scaffold and Evaluation
Rat	Rotator cuff injury model (Yea et al., 2020)	Small size, low cost; suitable for early-stage biomaterial screening due to easy surgery and handling	Quadruped support differs from humans; shoulder biomechanics differ significantly	Applicable for early screening; e.g., 4–8 weeks histological grading
	Achilles tendon injury model (Dong et al., 2024)	Low cost and mature technique; widely used in tendon injury models	Difficult to quantify gait and kinematic changes due to quadruped support; limited in studying chronic degeneration	Histological score ∼52% at 4 weeks, 263 μm scar zone; bone tunnel widening by ∼8–13%, tendon–bone failure rate ∼30.7%
Rabbit	Rotator cuff injury model (Wang et al., 2022)	Shoulder anatomy resembles humans (glenoid, deltoid insertion); allows dynamic motion testing	Anatomical differences: rabbit rotator cuff muscle group differs in orientation; shoulder kinematics differs from humans	Histology score ∼54% at 8 weeks; post-op fibrosis resolved by 12 weeks. Micro-CT shows clear bone–tendon integration and good material compatibility
	ACL reconstruction model (Liu et al., 2017)	Anatomical similarity to humans; femur-tibia structure is easy to operate on and facilitates standardised positioning and imaging	Four-legged motion patterns limit biomechanical transferability; steep learning curve in surgical manipulation	Micro-CT and histological scoring show good integration at 4–8 weeks; ECM remodelling rate >30%, suitable for biological material validation
	Patellar-patellar tendon complex injury model (Tang et al., 2020)	Strong translational relevance: includes femur, bone tunnel, and tendon grafts, simulating TBI repair. Suitable for tensile testing and histological evaluation	TBI model is complex; difficult to control tension and insertion depth; limited motor ability post-op; technically demanding	12-week studies show fibrocartilage-like tissue formation; histology reveals clear zone stratification and collagen alignment
Sheep	Rotator cuff injury model (Sensini et al., 2025)	Shoulder anatomical structure and muscle size are close to humans; suitable for biomechanical evaluation	Not widely used in standardised models; mostly suitable for large-animal SEM or scaffold implantation studies, with limited quantitative gait analysis	Applied in few scaffold studies; shoulder structure allows moderate material loading; limited in functional scoring; suitable for large constructs

### 2.4 Current clinical methods for tendon-bone interface injury repair and their limitations

Currently, the primary method for repairing complete tendon-bone interface tears clinically is surgical intervention to reattach the torn tendon (or ligament) to the bone (Yang et al., 2023). Commonly used techniques include direct suture or the use of grafts (Kamalitdinov et al., 2020). However, these traditional methods have significant limitations, the core issue being their inability to reconstruct the intricate gradient structure and biological functional integration of the native TBI. Whether through direct suture or graft fixation, a structural connection interface is typically formed between the tendon/graft and bone that is relatively abrupt and lacks an intermediate transition zone (Zhang et al., 2023c). This structural mismatch leads to the following key limitations:

Stress Concentration and High Re-tear Risk: New stress concentration points occur at the surgical repair interface, significantly increasing the risk of re-tear (Wang C. et al., 2024). For instance, a meta-analysis of rotator cuff repair for large tears showed a re-tear rate of approximately 50% even with double-row suture techniques (Villarreal-Villarreal et al., 2021). This highlights the limitations of traditional repair methods in withstanding high tensile loads. ACL reconstruction faces similar challenges. For example, a study comparing different grafts for ACL reconstruction found re-tear rates of 7.6%, 13.3%, and 8.7% for autograft quadriceps tendon, hamstring tendon, and bone-patellar tendon-bone, respectively (Ashy et al., 2023). This is primarily attributed to stress concentrations resulting from poor healing at the graft-bone tunnel interface.

Insufficient Biological Healing: At the surgical repair interface following TBI injury, fibrous scar tissue is often formed instead of a functional tendon-bone attachment site possessing the structural characteristics and excellent mechanical properties of native TBI (Vervaecke et al., 2022). A histological case study of patients 4 months post-ACL reconstruction revealed that portions of the graft-bone tunnel interface were still predominantly fibrotic scar tissue, lacking the gradual transition structure characteristic of the native tendon-bone interface (Lazarides et al., 2015).

Graft-related Issues: When using autografts, there is a risk of donor site morbidity (e.g., pain, functional impairment) (James et al., 2023). For example, using hamstring tendons for ACL reconstruction can lead to postoperative posterior knee pain and weakness in knee flexion (Widner et al., 2019). While the use of allografts may face issues such as immune rejection, risk of disease transmission, and slower graft integration (Kyung, 2019). A prospective randomized clinical study reported that patients undergoing allograft tendon transplantation experienced a longer duration of fever postoperatively compared to autograft, potentially due to immune rejection (Sun et al., 2009).

**FIGURE 2:**
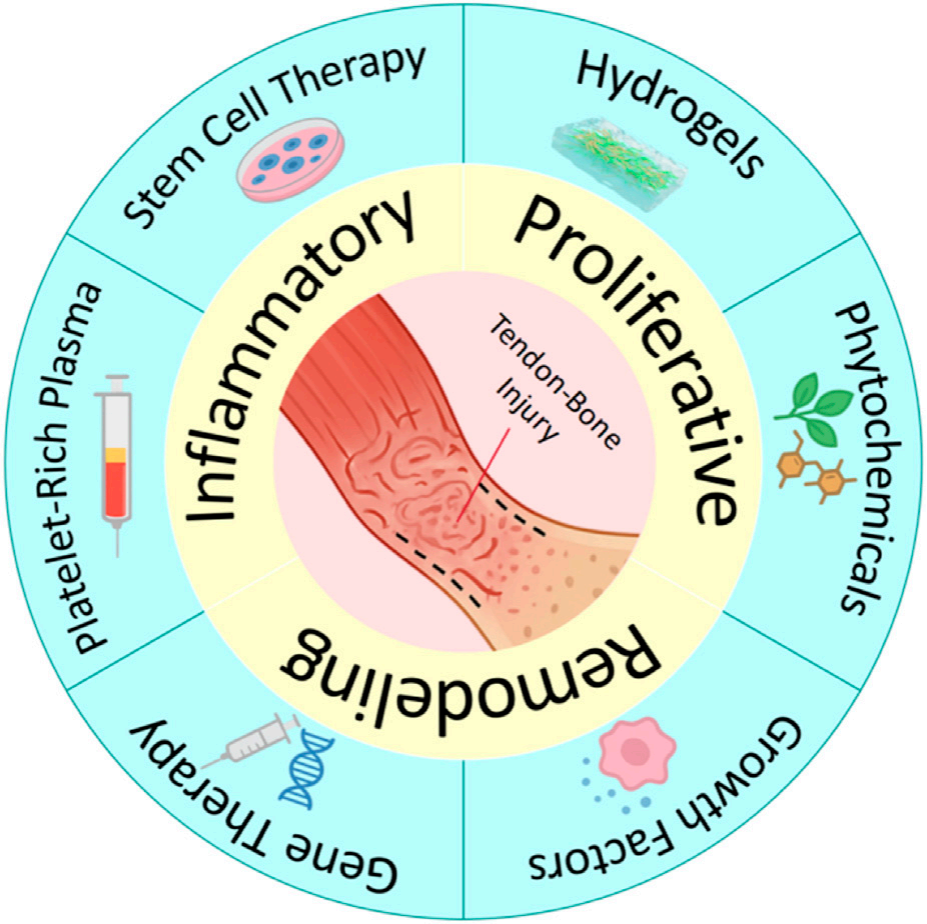
Repair process and therapeutic strategies for tendon–bone injuries.

To address the aforementioned challenges, researchers have developed several strategies to augment traditional methods and enhance tendon-bone repair. These include local injections of platelet-rich plasma (Zheng et al., 2019), stem cells (Wang Y. et al., 2024), and bioactive factors (Lei et al., 2021). Figure 2 show the repair process and therapeutic strategies for tendon-bone injuries. Furthermore, commercial patches designed to enhance tendon-bone repair are already available on the market, such as acellular dermal matrices (REPORTS, 2025), porcine small intestinal submucosa (SIS) matrices, and bovine Achilles tendon-derived collagen patches. However, these strategies lack spatial localisation and structural templating, struggle to maintain stable bioavailability, fail to provide immediate mechanical support, or are unable to restore the continuous gradient transition structure of the TBI after tendon-bone injury. This can lead to stress concentrations and subsequent re-injury. Therefore, developing novel repair strategies capable of mimicking the native TBI gradient structure, promoting functional tissue regeneration, and improving mechanical integration is a significant challenge and urgent need currently faced in the field of tendon-bone interface regenerative medicine.

## 3 Design principles and biomimetic strategies for gradient biomimetic scaffolds

### 3.1 Design principles and biomimetic strategies for physical structure gradient scaffolds

A prominent characteristic of the native TBI is its continuous gradient variation in physical structure, including gradual changes in tissue stiffness, fiber alignment, and microscopic pore structure. This feature is crucial for achieving smooth stress transfer from flexible tendon to rigid bone (Aghaei et al., 2021). The design of physical structure gradient scaffolds aims to mimic these characteristics, thereby more accurately simulating the microenvironment of native tissue, providing suitable conditions for cell attachment, proliferation, and differentiation, and consequently guiding ordered tissue regeneration. Designing physical structure gradient scaffolds should adhere to two core principles: ① Principle of Continuous Gradient: Simulating the stress dissipation mechanism of native tissue and avoiding stress concentration caused by abrupt interfaces through smooth transitions in porosity, fiber alignment, and mechanical modulus; ② Principle of Regional Biomimicry: The gradient scaffold should mimic the characteristic physical structures of the corresponding native tissues (tendon, fibrocartilage, bone) in different regions.

Strategies for achieving physical structure gradients primarily include: ① Pore Structure Gradient: From tendon to bone in native TBI, there are gradient changes in porosity and pore size (Tits et al., 2021). Existing research has designed and fabricated gradient scaffolds with porosity and/or pore size gradually increasing from the tendon end towards the bone end (Lang Bai et al., 2024). Smaller pores and lower porosity (tendon end) are beneficial for mimicking dense collagen fiber bundles, which may guide cell alignment along specific directions to influence cell behavior and provide certain initial mechanical support (Dong et al., 2024). In contrast, larger pores and higher porosity (bone end) are considered favorable for promoting cell migration and proliferation (especially osteoblasts and vascular endothelial cells), as well as subsequent vascularization, bony ingrowth, and mineral deposition (Dai et al., 2024). Gradient pore structure not only regulates cell behavior but also directly affects the overall mechanical properties of the scaffold and the transport efficiency of internal substances (nutrients, metabolites). ② Fiber Orientation Gradient: Collagen fibers in tendon tissue exhibit a high degree of parallel alignment to accommodate uniaxial tensile load (Tits et al., 2023). However, in the TBI transition zone and bone zone, fiber arrangement becomes irregular or reticular interwoven. Researchers have utilized techniques such as electrospinning to fabricate scaffolds with fiber orientation gradients: At the end mimicking tendon, nanofibers are highly parallelly aligned to guide directed growth and ECM deposition of tenocytes (Bianchi et al., 2022) and impart excellent axial tensile strength to the scaffold (Zhang et al., 2012; Erisken et al., 2013); while transitioning towards the bone end, fiber arrangement can gradually become random or form specific angles to mimic the structural characteristics of fibrocartilage and bone (Bianchi et al., 2022), adapt to more complex stress states, and promote three-dimensional cell distribution. ③ Mechanical Property Gradient: The gradient changes in physical structure directly lead to a gradient in the scaffold’s mechanical properties (e.g., elastic modulus, stiffness). Researchers have achieved a natural modulus gradient spanning from the flexible tendon end to the rigid bone end by controlling the density and orientation of electrospun fiber (Xiong et al., 2022), or by varying infill density/patterns in 3D printing (Bittner et al., 2019). This mechanical gradient is crucial for reducing stress concentration and promoting mechanical signal transduction.

### 3.2 Design principles and biomimetic strategies for chemical composition gradient scaffolds

Chemical composition gradient is another important strategy in gradient biomimetic scaffold design. Its core principle lies in mimicking the continuous change in chemical composition of native TBI, transitioning from an organic matrix-dominated region to an inorganic mineral-dominated region (Pitta et al., 2023), thereby promoting interface region-specific cellular responses and tissue integration. The most significant chemical change across the native TBI, from tendon to bone, is the marked difference in the degree of mineralization (primarily hydroxyapatite, HA) (Chen et al., 2023). Therefore, chemical composition gradient design typically utilizes composite materials of polymers and inorganic minerals, spatially controlling the type and content of inorganic minerals to achieve gradient variations, promoting tissue regeneration and mineralization.

Based on the source of chemical materials, chemical biomimetic scaffolds can be classified into: ① Polymer Material Gradient: Polymers with different biological properties and degradation rates can be selected. For example, one end of the scaffold can use materials favorable for tenocyte attachment and growth (such as Type I collagen, silk fibroin), while the other end can incorporate or entirely consist of materials more affinity for osteoblasts and supporting mineralization (such as calcium phosphate-doped polymers) (Geng et al., 2024). ② Inorganic Component Gradient: Mimicking the gradient in the degree of mineralization within the TBI is one of the core aspects of chemical composition gradients. Typically, inorganic ceramic phases such as hydroxyapatite, tricalcium phosphate, or bioactive glass are introduced at the bone end of the scaffold, with their concentration increasing from zero or low concentration at the tendon end to high concentration at the bone end (Gao et al., 2023; Chen Y. et al., 2024; Cao et al., 2020; Bayrak et al., 2017). These inorganic components possess excellent osteoconductivity and osteoinductivity, significantly promoting the differentiation of osteoblasts and the deposition of mineralized matrix, while also effectively increasing the hardness and modulus of that region of the scaffold (Fang et al., 2019; Sun et al., 2022). ③ Native ECM Component Gradient: A more refined biomimetic strategy is to mimic the gradient distribution of specific ECM molecules within the TBI, such as Type I collagen (enriched in the tendon zone), Type II collagen (enriched in the fibrocartilage zone). (Taylor et al., 2020). Although this technique is more technically challenging, some studies have attempted to construct such gradients by methods like regional coating or loading of different ECM extracts (Olvera et al., 2020).

### 3.3 Design principles and biomimetic strategies for biological signal gradient scaffolds

The formation, maintenance, and repair processes of the native tendon-bone interface are regulated by a complex biological signaling network. The spatio-temporal specific distribution of signaling molecules within this network is crucial for precisely regulating the recruitment, proliferation, migration, and directed differentiation of stem/progenitor cells, ultimately promoting the formation of an integrated structural-functional transition zone (Dang et al., 2022). Constructing biomimetic biological signal gradients (particularly growth factor gradients) in tissue engineering scaffolds is considered one of the key strategies for achieving functional TBI regeneration.

The core design principles for biological signal gradient scaffolds include: ① Precise spatio-temporal mimicry of the distribution patterns of key signaling molecules during native TBI development or repair processes. ② Controlled sequential release: Native TBI repair is a multi-stage process, with different signaling molecules playing roles at different time points (Adler, 2019).

To achieve the aforementioned design principles, researchers have employed various biomimetic strategies to construct biological signal gradients: ① Growth Factor/Cytokine Gradient: Based on the cellular differentiation requirements in different TBI zones, corresponding growth factors can be loaded into different regions of the scaffold. For example, factors promoting tendon differentiation, such as Growth Differentiation Factor-7 (GDF-7) and Transforming Growth Factor-beta 3 (TGF-β3), can be loaded at the tendon end (Han et al., 2020); factors promoting cartilage differentiation, such as TGF-β1/β3 and Bone Morphogenetic Protein-2 (BMP-2), can be loaded in the fibrocartilage region (Wang L. et al., 2024; Dorcemus et al., 2021); and factors promoting osteogenic differentiation and vascularization, such as BMP-7 and Vascular Endothelial Growth Factor (VEGF), can be loaded at the bone end (Rambhia et al., 2024; Godoy-Gallardo et al., 2020). ②Small Molecule Drug Gradients: Based on the specific requirements of different stages of tendon-bone injury repair, corresponding drugs can be loaded into different regions of the scaffold. For example, anti-inflammatory drugs, such as dexamethasone, can be loaded throughout the scaffold (Majrashi et al., 2023); while drugs that accelerate vascularisation, such as deferoxamine, can be loaded specifically within the bone region (Wang R. et al., 2024). ③Bioactive Ion Gradients: As inherently stable substances, bioactive ions can be directly incorporated into the scaffold. For instance, the addition of magnesium ions (Mg^2+^) to bioinks allows for sustained release during scaffold degradation, promoting bone integration and vascular ingrowth (Safiaghdam et al., 2023). Similarly, incorporating silicon or strontium ions into polymer solutions can induce osteoblast proliferation and modulate osteoclast activity (Lin et al., 2022). ④ Signal Gradient Construction and Controlled Release: The key to realizing biological signal gradients lies in precise spatial localization and controlled release kinetics (Freeman et al., 2020; Wang et al., 2020; Xu et al., 2020). Common methods include encapsulating growth factors within nanoparticles or hydrogels and then distributing them in a gradient manner within the scaffold to achieve sustained and controlled release rates, utilizing core-shell or multi-layer structures in electrospinning to load different factors separately, and gradient mixing of growth factors into bioinks during 3D printing for fabrication (Freeman et al., 2020; Wang et al., 2020; Xu et al., 2020). However, maintaining growth factor activity, avoiding burst release, and achieving long-term, ordered release that matches the temporal progression of tissue regeneration remain current challenges (Zimmerling et al., 2024). ⑤ Gene/Nucleic Acid Gradient: As a persistent mode of signal regulation, gradient loading of nucleic acid molecules such as mRNA encoding specific growth factors within the scaffold, to induce local cell expression of desired proteins through gene delivery, is also an emerging research direction (Zaitseva et al., 2019; Zaitseva et al., 2020).

### 3.4 Design principles and biomimetic strategies for time-gradient biomimetic scaffolds

Tendon-bone repair is a highly dynamic biological process involving three distinct phases: inflammation, proliferation, and remodelling (Xu M. et al., 2024). Each phase involves specific cell types, signalling pathways, and tissue morphogenesis processes, which collectively determine the structural regeneration and functional recovery of the nascent tissue (Xu Z. et al., 2024). Therefore, incorporating a temporal dimension into biomimetic scaffold design, ensuring that scaffold properties are coordinated with the rhythm of tendon-bone repair, is crucial for achieving “process biomimicry.” The design principles for time-gradient biomimetic scaffolds primarily focus on two aspects (Killian, 2022): Ordered release of signalling molecules: providing appropriate signalling factors or microenvironmental regulators at different stages of repair; and (Dang et al., 2022) Dynamic mechanical matching: ensuring that the scaffold’s mechanical strength, degradation rate, and physical properties are synchronised with the requirements of tissue reconstruction, thereby optimising cell-material interactions and promoting functional tissue integration.

To achieve these design principles, researchers have employed various biomimetic strategies to construct scaffolds that address the temporal requirements of tendon-bone repair: ①Sequential Delivery Systems: These systems aim to precisely guide the repair rhythm by controlling the release of growth factors, immunomodulatory molecules, or small molecule drugs (Chen T. et al., 2024). For example, incorporating microspheres encapsulating various drugs, such as dexamethasone, deferoxamine, and Kartogenin, into the scaffold matrix allows for differential drug release based on the microsphere material and structure (Zhang et al., 2024; Pisani et al., 2023; Ji et al., 2022). This approach enables the suppression of excessive inflammation in the early stages of repair while promoting cell proliferation and tissue generation in the later stages (Zhang et al., 2024; Pisani et al., 2023; Ji et al., 2022). ② Multilayered Sustained-Release Structures: This strategy utilises multilayered or core-shell structures to achieve sequential release of repair signals. For instance, in a core-shell structure, anti-inflammatory drugs can be loaded in the shell, while growth factors are encapsulated in the core (Zhao W. et al., 2024). Alternatively, a three-layered scaffold can be loaded with inflammatory factors, chondrogenic factors, and osteogenic factors from the outer to inner layers, reflecting not only spatial hierarchy but also a temporal sequence from immunomodulation to tissue differentiation (Lang Bai et al., 2024). ③Smart Responsive Materials: These materials respond to endogenous stimuli such as pH, temperature, and enzymes, or exogenous stimuli like light, electricity, and magnetism, creating a “self-regulating” temporal response mechanism (Singh et al., 2024). This allows for precise control of cell behaviour and signal regulation at different stages of repair (Singh et al., 2024). For example, the low pH and high reactive oxygen species (ROS) levels characteristic of the inflammatory phase can trigger the specific release of anti-inflammatory drugs from a hydrogel, modulating the tendon-bone microenvironment (Qi et al., 2024). In later stages, as matrix metalloproteinase activity increases, enzyme-responsive smart materials can degrade on demand, gradually transferring mechanical stress to the newly formed tissue (Xing et al., 2023).

The key contents of the design principles and biomimentic strategies for the aforementioned four types of gradient biomimetic scaffolds are summarized in Table 3.

**TABLE 3:** Design principles and biomimetic strategies for gradient biomimetic scaffolds.

Scaffold Type	Design Principle	Biomimetic Strategy	Biomimetic Target	Key Materials	Core Function and Significance
Physical Structure Gradient	Continuous Gradient/Regional Biomimicry	Porosity Gradient (Dai et al., 2024; Dong et al., 2024; Lang Bai et al., 2024)	Simulate porosity/pore size changes from dense tendon to porous bone	Porous polymers (e.g., PCL, silk fibroin)	Small pores guide aligned cell growth on tendon side; large pores promote cell migration, vascularization, and bone ingrowth on bone side
		Fiber Alignment Gradient (Bianchi et al., 2022)	Simulate fiber structures from highly aligned in tendon to random in bone	Nano/microfibres (e.g., PCL, PLA)	Aligned fibers provide axial tensile strength and guide tendon cell growth; random fibers adapt to stress and aid 3D integration
		Mechanical Property Gradient (Xiong et al., 2022; Bittner et al., 2019)	Simulate elastic modulus transition from flexible tendon to rigid bone	polymers (e.g., PCL, TPU)	Enable smooth stress transfer, avoid stress concentration, reduce re-tear risk, and provide mechanical cues
Chemical Composition Gradient	Organic to Inorganic Matrix Transition	Polymer Material Gradient (Geng et al., 2024)	Simulate compositional gradient from tendon to bone in natural TBI	Tendon side: Type I collagen, silk fibroin; Bone side: CaP-doped polymers, HA	Promote tenogenic and osteogenic cell attachment and proliferation via compositional gradients, aiding TBI regeneration and integration
		Inorganic Composition Gradient (Gao et al., 2023; Chen et al., 2024a; Cao et al., 2020)	Simulate continuous mineralization (e.g., hydroxyapatite) in TBI	HA, TCP, BG	Promote osteogenic differentiation and mineral deposition, enhancing scaffold osteointegration and mechanical strength
		Natural ECM Component Gradient (Taylor et al., 2020; Olvera et al., 2020)	Simulate regional distribution of specific ECMs in TBI	Type I Collagen, Type II Collagenetc.	Provide targeted adhesion sites for region-specific cells to guide realistic tissue regeneration
Biological Signal Gradient	Spatial and Temporal Distribution of Key Signaling Molecules	Growth Factor/Cytokine Gradient (Han et al., 2020; Wang et al., 2024d; Dorcemus et al., 2021; Rambhia et al., 2024; Godoy-Gallardo et al., 2020)	Simulate spatial-temporal distribution of key signals in TBI repair	TGF-β3, BMP-2, GDF-7, VEGF	Precisely guide stem cell differentiation towards tendon, cartilage, and bone by region-specific signal delivery
		Small Molecule Drug Gradient (Majrashi et al., 2023; Wang et al., 2024e)	Deliver drugs to modulate local microenvironment	Dexamethasone Deferoxamine	Modulate microenvironment and control cellular behavior via region-specific drug loading
		Bioactive Ion Gradient (Safiaghdam et al., 2023; Lin et al., 2022)	Sustained ion release promoting tissue regeneration	Mg^2+^, Sr^2+^, Si^2+^	Long-term modulation of cell behavior through sustained ion release
		Gene/Nucleic Acid Gradient (Zaitseva et al., 2019; Zaitseva et al., 2020)	Gene delivery to stimulate local expression of required growth factors	mRNA/pDNA	Enable long-term and stable signal supply, overcoming short half-life of growth factors
Temporal Gradient	Sequential Signal Release/Dynamic Mechanical Matching	Sequential Delivery System (Zhang et al., 2024; Pisani et al., 2023; Ji et al., 2022)	Simulate release pattern of growth factors in TBI repair	Microsphere-loaded dexamethasone, deferoxamine, kartogenin	Achieve differentiated release by tuning microsphere structure and material
		Multi-layered Sustained-Release Structure (Lang Bai et al., 2024; Zhao et al., 2024b)	Spatiotemporal signal release for sequential tissue repair	Multilayer or core-shell structured scaffolds	Temporal-spatial signaling for immune modulation to tissue differentiation
		Smart Responsive Materials (Qi et al., 2024; Xing et al., 2023)	Stimuli-responsive release for self-regulated tissue healing	pH/temperature/enzyme/light/electric/magnetic-responsive materials	Autoregulated temporal response to microenvironmental cues

## 4 Materials for gradient biomimetic scaffolds in tendon-bone healing

The successful fabrication of gradient biomimetic scaffolds is highly dependent on the properties of the selected materials, as appropriate materials can impart specific biological functions to the scaffold. Ideal materials should not only possess good biocompatibility, suitable mechanical properties, and a controllable degradation rate, but also be amenable to being shaped into precisely graded structures using existing manufacturing techniques (Wang et al., 2025; Zhong et al., 2025). Currently, various biomaterials commonly used for constructing gradient scaffolds and their functions are described below.

### 4.1 Natural polymers

Natural polymers are highly favored in tissue engineering scaffold construction due to their wide availability, biodegradability, and similarity to ECM components (Singh S. et al., 2023). They often contain cell-recognition sites that promote cell adhesion, proliferation, and differentiation, and their tunable molecular structures and physicochemical properties provide a basis for constructing compositional or functional gradients (Di et al., 2023). Several natural polymers currently used for tendon-bone healing are described below:

Collagen: As the most abundant protein in connective tissue, collagen (especially type I collagen) is an ideal choice for mimicking the ECM of tendons and bone (Dewle et al., 2020; Vijayalekha et al., 2023; Selvaraj et al., 2024). The plasticity of its molecular structure allows for the construction of collagen-based scaffolds through various strategies. On one hand, physicochemical gradients can be created by controlling collagen concentration, crosslinking degree, or composite formation with other materials. This not only affects the macroscopic properties of the scaffold but also provides topographical cues for cells, regulating cell behavior (Dai et al., 2024). On the other hand, scaffolds with varying collagen content in different regions can be fabricated through layer-by-layer deposition or segmented construction, achieving gradients in mechanical properties and degradation rate (Zhong et al., 2025). Furthermore, collagen matrices can serve as carriers for the release of bioactive molecules, further guiding the specific regeneration of interface tissues (Pugliese et al., 2023). However, when collagen is used to construct gradient biomimetic scaffolds, its relatively weak mechanical properties and rapid degradation rate often require optimization through crosslinking, material compositing, or the use of recombinant collagen to meet the demands of TBI repair, particularly in load-bearing areas (Dai et al., 2024; Zhang Z. et al., 2023).

Silk Fibroin (SF): Derived from silkworm silk, silk fibroin exhibits excellent biocompatibility, controllable degradation, and strong mechanical properties (Hamad et al., 2020), demonstrating significant advantages in constructing TBI gradient biomimetic scaffolds that require load-bearing capacity. Its unique molecular structure, particularly the β-sheet crystalline structure, can be precisely controlled through processing or post-treatments such as water vapor annealing and methanol/ethanol exposure, thereby regulating its mechanical strength and degradation rate (Lin et al., 2013; Li et al., 2023). Utilizing this characteristic, silk fibroin scaffolds with gradient changes in mechanical properties and degradation rates can be prepared to meet the regeneration needs and mechanical transmission requirements of different regions of the TBI, from the flexible tendon end to the rigid bone end. In addition, the SF surface is easily functionalized, effectively guiding cell adhesion, migration, and differentiation (Liu et al., 2024). Simultaneously, its composite with inorganic materials such as hydroxyapatite can effectively mimic the mineralization gradient of the TBI, promoting osseointegration (Geng et al., 2024). Despite its clear advantages, the potential for immune responses related to its source and purification should be considered in its application.

Chitosan: As a positively charged natural polysaccharide, chitosan possesses good biocompatibility, biodegradability, antibacterial activity, and wound-healing capabilities, making it attractive in the field of TBI repair (Ahmed and Ikram, 2016; Deng et al., 2024). The amino and hydroxyl groups in its structure facilitate chemical modifications such as grafting, crosslinking, and the introduction of functional groups, allowing for fine-tuning of its physicochemical properties and bioactivity (Elnaggar et al., 2024). At the same time, the degradation rate and mechanical properties can be controlled by adjusting chitosan deacetylation or molecular weight (Feng et al., 2024), thereby constructing polysaccharide gradient scaffolds. Its unique positive charge can be used for electrostatic adsorption or delivery of negatively charged biomolecules such as nucleic acids (Dey et al., 2020). Furthermore, the pH responsiveness of chitosan has been explored for constructing intelligent biomimetic materials, such as triggering the gradient release of active substances in specific microenvironments. Compositing chitosan with inorganic materials such as hydroxyapatite effectively mimics the critical mineralization gradient of the TBI, thereby promoting osseointegration (Chen et al., 2019). However, the relatively weak mechanical properties of chitosan (especially in the liquid state) make it difficult to meet the needs of the TBI, particularly in the mechanical transition zone and tendon end. Therefore, the stability of chitosan should be carefully considered when constructing load-bearing gradient structures.

Other natural polymers, such as alginate and hyaluronic acid, also have potential applications in constructing tendon-bone gradient hydrogel scaffolds due to their unique physicochemical properties such as hydrogel-forming ability, but their extremely low mechanical strength limits their application in TBI repair. This shortcoming in mechanical performance is a common problem that many natural polymer materials need to overcome when addressing TBI repair needs. In summary, although natural polymers have good biocompatibility and can mimic some ECM components, they generally suffer from insufficient mechanical properties, difficult-to-control degradation rates, batch-to-batch variability, and immune risks, making it difficult to meet the needs of load-bearing TBI gradient repair alone. Therefore, with the development of material compositing and functional modification technologies, current research mainly focuses on compositing them with inorganic materials such as hydroxyapatite and bioactive glass, or synthetic polymers such as Polycaprolactone (PCL) and Poly (lactic-co-glycolic acid) (PLGA), and combining chemical modification and crosslinking optimization to improve their mechanical properties and structural stability, making their functional expansion in gradient scaffolds towards the transition zone and even the bony region possible.

### 4.2 Synthetic polymers

Synthetic polymers offer unique advantages in constructing biomimetic scaffolds for the tendon-bone interface due to their controllable chemical structures, predictable degradation behavior, and stable mechanical properties (Xu and Song, 2020). These inherent characteristics make them key components for achieving complex biomimetic structures with specific mechanical transmission, degradation timing, and structural organization requirements. Several synthetic polymers currently used for tendon-bone healing are described below:

Polycaprolactone (PCL): PCL is a biocompatible polyester whose most notable feature is its slow degradation rate (typically lasting several years), providing long-term mechanical support for the slowly healing tendon-bone interface (Faezeh et al., 2017). Its low melting point (approximately 60 °C) (Aoyama et al., 2021) facilitates composite formation with other materials, such as inorganic ions, or bioactive molecules with certain thermal sensitivity requirements. PCL exhibits excellent processability and can be easily used with advanced manufacturing techniques such as multi-nozzle electrospinning or 3D printing to construct PCL-based scaffolds with gradients in porosity, fiber diameter, or composite ratio with inorganic materials such as hydroxyapatite (Zhao et al., 2022). For example, by precisely controlling the extrusion rate or deposition density of the PCL solution/melt, PCL-based scaffolds with graded pore structures and local mechanical properties can be fabricated to mimic the structural transition of the TBI(77). However, the bioinertness and hydrophobic surface of PCL may be unfavorable for effective cell adhesion and function. Its inherently low mechanical modulus makes it difficult to mimic the high stiffness of natural bone alone, and its slow degradation may not always match the rate of tissue regeneration and remodeling (Chen et al., 2022a).

Polylactic Acid (PLA) and Poly (lactic-co-glycolic acid) (PLGA): As FDA-approved biodegradable polyesters, PLA and PLGA are widely used due to their good biocompatibility and degradability via metabolic pathways in the body (Swider et al., 2018; Yang et al., 2024). The core advantage of PLGA lies in its degradation rate, which can be precisely controlled by adjusting the ratio of lactic acid (LA) to glycolic acid (GA), making it an ideal material for constructing scaffolds with different degradation rates (He et al., 2015). This gradient design aims to better match the tissue regeneration rate and mechanical requirements of the TBI, where faster degradation is needed at the bone end and slower degradation at the tendon end. For example, scaffolds with varying degradation behaviors can be prepared by blending PLGA with different LA/GA ratios (Díaz et al., 2017). PLA, which is typically slower to degrade and has higher mechanical properties such as hardness and strength but may be more brittle, is suitable for applications requiring more sustained support (Chou et al., 2016). In addition, PLA and PLGA are often composited with hydroxyapatite and other materials (Wei et al., 2022; He et al., 2018) to introduce mineralization gradients, enhance osseointegration, and optimize mechanical properties. However, the acidic products released during their degradation (Elmowafy et al., 2019) may not only trigger aseptic inflammatory reactions locally but also adversely affect cell function and matrix deposition, and even accelerate the disintegration of the material itself, which is a concern that needs to be considered in applications.

Other synthetic polymers also provide unique performance options for TBI gradient scaffolds. For example, polyurethane (PU) offers excellent elasticity and toughness (Xu and Hong, 2022), enabling it to mimic the nonlinear mechanical response and energy dissipation characteristics of soft tissues such as tendons. By controlling the hard/soft segment chemical structure, elastic modulus gradient scaffolds can be constructed, but the long-term biostability of the formulation and the safety of degradation products still need to be considered (Ma et al., 2011; Mi et al., 2018). In summary, although synthetic polymers exhibit good structural designability and performance tunability in the construction of tendon-bone gradient biomimetic scaffolds, there are still certain limitations. For example, their inherent bioactivity is low, often requiring surface modification, composite formation with functional factors, or blending with natural polymers to enhance cell affinity. At the same time, some synthetic materials may produce acidic intermediates during degradation, causing potential irritation to the local microenvironment. Furthermore, how to achieve precise matching of material degradation behavior with the regeneration rhythm of different tissues at the tendon-bone interface still requires further systematic exploration.

### 4.3 Inorganic bioactive materials

Inorganic materials, primarily rigid functional materials composed of non-carbon-based minerals, are indispensable components for mimicking the mineralization gradient from cartilage to bone and for bearing the mechanical load at the bone end of the native TBI (Zhong et al., 2025). They not only provide the necessary rigidity and structural support but also regulate cell behavior through ion release, endowing the scaffold with osteoconductivity, osteoinductivity, and even pro-angiogenic activity (Dittler et al., 2019; Bi et al., 2012). Currently, commonly used inorganic bioactive materials for gradient biomimetic scaffolds include calcium phosphate ceramics and bioactive glasses.

Calcium Phosphate Ceramics: Due to their similarity to the mineral components of natural bone, calcium phosphate ceramics provide a crucial osteoconductive interface for gradient biomimetic scaffolds. These ceramics mainly include hydroxyapatite (HA), tricalcium phosphate (TCP), and their composites (Mate-Sanchez de Val et al., 2014). Hydroxyapatite (HA) has a chemical composition highly similar to bone mineral and exhibits excellent biocompatibility and osteoconductivity, providing a favorable environment for cell adhesion and proliferation and guiding bone tissue ingrowth (Zhou et al., 2019). Furthermore, its slow degradation rate provides long-term structural stability and a durable osseointegration interface, making it commonly used in the bone end region of gradient scaffolds (Gervaso et al., 2016). In contrast, β-tricalcium phosphate (β-TCP) has a faster degradation rate (Tran et al., 2025). The Ca^2+^ and PO_4_^3−^ ions released during its degradation can not only be utilized by cells but are also considered to have some osteoinductive activity, stimulating the proliferation and differentiation of osteoblasts (Tran et al., 2025). To more precisely control performance to match the complex gradient requirements of the TBI, biphasic calcium phosphate (BCP), a mixture of HA and β-TCP, is widely used. By precisely controlling the ratio of HA to β-TCP, the overall degradation rate and bioactivity intensity of BCP can be effectively tuned, achieving a smooth transition from stable to faster degradation (LeGeros et al., 2003). Integrating BCP into a polymer matrix (such as PCL) is an effective strategy for constructing TBI gradient scaffolds. For example, Woraporn et al. successfully used 3D printing to construct a BCP/PCL scaffold that spatially guided the region-specific differentiation of MSCs towards the osteogenic lineage (Supphaprasitt et al., 2022), strongly demonstrating the feasibility of mimicking the native TBI microenvironment and guiding functional regeneration by controlling the BCP composition.

Bioactive Glasses (BGs): BGs can provide a functional combination of both osteoinductive and angiogenic activities (Che et al., 2022). Through surface reactions in body fluids, BGs dissolve and release ions such as Si^4+^, Ca^2+^, and PO_4_^3-^, which then form a hydroxycarbonate apatite (HCA) layer, enabling rapid chemical bonding with the host bone and achieving rapid and stable osseointegration (Che et al., 2022). More importantly, the Si^4+^ and Ca^2+^ ions released by BGs not only upregulate osteogenic gene expression but also significantly stimulate VEGF production, promoting angiogenesis to provide nutrients, oxygen, and remove metabolic waste (Font Tellado et al., 2021). Currently, integrating BGs into scaffolds in a gradient distribution has been proven to simultaneously promote osteogenic differentiation and vascularization. For example, one study successfully prepared a porous nanocomposite scaffold by integrating micro-nano bioactive glass (MNBG) into a PLGA porous scaffold. This scaffold effectively promoted osteogenic differentiation of mesenchymal stem cells and angiogenesis of endothelial cells *in vitro*, demonstrating its potential for tendon-bone regeneration (Tian et al., 2019).

Inorganic bioactive materials not only play a crucial structural and mechanical support role in tendon-bone gradient biomimetic scaffolds but also participate in the regeneration process of bone-end tissues through their ion-mediated and interface-induced mechanisms. However, the brittleness and low toughness of inorganic materials, as well as the limited interfacial compatibility with polymer matrices, remain technical bottlenecks that restrict their independent or high-proportion use. Future efforts should focus on developing highly dispersible inorganic nano-components, interface control technologies, and intelligent responsive inorganic functional systems to provide more integrated material solutions for achieving precise biomimetic reconstruction of the tendon-bone interface.

A comparative summary of the aforementioned three types of materials used for constructing gradient biomimetic scaffolds is presented in Table 4.

**TABLE 4:** Comparison of polymeric materials in gradient biomimetic scaffolds.

Material Type	Representative Materials	Main Advantages	Main Disadvantages	Gradient Function Suitability Example
Natural Polymers	Collagen, Silk Fibroin, Chitosan, Alginate, Hyaluronic Acid	1. Excellent biocompatibility and bioactivity: natural ECM components can support cell adhesion and guide regeneration. 2. Biodegradable and resorbable, often enzymatically: suitable for temporary scaffolds. 3. Good processability and functionalization potential: supports design of bionic structures	1. Weak mechanical properties: difficult to meet TBI load-bearing needs, especially tendon zone. 2. Unstable degradation: enzymatic degradation may cause local mismatch or inflammation. 3. Difficult to control source and batch consistency: poor mechanical reliability. 4. Low surface stiffness: not suitable for mineralisation-rich bone zones	Used for constructing collagen or chondrogenic zones in gradient scaffolds (e.g., tendon end or fibrocartilage)
Synthetic Polymers	Polycaprolactone (PCL), Polylactic Acid (PLA), Polylactic-co-glycolic Acid (PLGA), Polyurethane (PU)	1. Stable and tunable mechanical properties: customisable to meet structural requirements. 2. Predictable degradation rates: adjustable according to needs (e.g., PLGA). 3. Good processing capability: supports 3D printing and electrospinning for fine control. 4. Abundant sources and relatively low cost	1. Poor bioactivity (inert to cells): limited cell adhesion or signal induction.2. Degradation may produce acidic by-products (e.g., PLA/PLGA), leading to inflammation. 3. Hydrophobic surfaces: low tissue affinity and cell permeability. 4. Single material may not simultaneously match tendon and bone needs	Used in constructing intermediate gradient transition zones (e.g., between tendon and bone)
Inorganic Bioactive Materials	Hydroxyapatite (HA), Tricalcium Phosphate (TCP), Bioactive Glass (BG)	1. Excellent osteoconductivity and osteoinductivity: structurally similar to bone, supports mineralization. 2. Provides stiffness and mechanical strength: used to reinforce weak structures. 3. Releases bioactive ions (e.g., Si^4+^, Ca^2+^) that promote bone and vascular regeneration. 4. Chemically stable in physiological environments	1. Brittle and poorly elastic: difficult to match soft tissues like tendons. 2. Difficult to degrade: may persist long-term *in vivo*. 3. Poor interfacial integration with soft tissue: mismatched mechanical properties. 4. Requires composite with polymers to enhance formability	Used to build bone zones: suitable for mineralised ends of scaffolds

### 4.4 Other materials

Besides the materials mentioned above, decellularized extracellular matrix (dECM) and metallic materials have also demonstrated unique potential in constructing gradient biomimetic scaffolds.

dECM is derived by removing immunogenic cellular components from tissues through chemical or physical methods, while preserving the complex composition, three-dimensional ultrastructure, and biomechanical properties of the native extracellular matrix, including collagen, elastin, glycosaminoglycans, proteoglycans, and various growth factors (Pati et al., 2014; Chen et al., 2020). Because dECM provides a rich source of biochemical signals and a tunable matrix microenvironment, constructing dECM scaffolds that mimic the gradient changes in ECM composition, structure, and mechanical properties of the native tendon-bone interface can effectively guide cell migration, differentiation, and matrix deposition, ultimately promoting the reconstruction of a functional tendon-bone interface. For example, multi-layered assembly of dECM derived from cortical bone, pubic symphysis, and Achilles tendon can directly simulate the structural gradient of the tendon-bone interface (Tang et al., 2020). Microfluidic techniques can be employed to create continuous growth factor concentration gradients within decellularized tendon scaffolds, precisely regulating stem cell differentiation and achieving a three-layered structure similar to the native tendon-bone interface (Lyu et al., 2020). Furthermore, mineral gradients can be successfully constructed in decellularized bone scaffolds through methods like EDTA demineralization or coating-controlled demineralization, mimicking the mineral gradient of the tendon-bone interface and effectively regulating cell differentiation (Zhou et al., 2020). However, the clinical translation of dECM gradient scaffolds still faces challenges such as batch-to-batch variations, potential immunogenicity, and the need for improved mechanical properties. Future research should focus on developing standardized dECM preparation processes, efficient gradient construction techniques, and multifunctional integration strategies to ultimately achieve the clinical application of dECM gradient biomimetic scaffolds in tendon-bone interface regeneration.

Metallic materials, with their excellent mechanical properties, machinability, and corrosion resistance, also offer unique advantages in constructing gradient biomimetic scaffolds (Chen et al., 2022b). Among them, titanium alloys, tantalum, and magnesium alloys have shown promise for tendon-bone gradient biomimetic scaffold fabrication due to their good biocompatibility (Saab et al., 2023; García-Robledo et al., 2024; Zou et al., 2023). For instance, porous tantalum scaffolds, with their high porosity and specific surface area, can promote bone ingrowth and can mimic the gradient changes of the tendon-bone interface by adjusting pore size gradients (Wang et al., 2023). 3D printing technology also offers the possibility of fabricating metallic scaffolds with complex geometries and gradient porosity. For example, titanium alloy scaffolds with gradient porosity and mechanical properties can be fabricated by controlling the deposition density of metal powder and the laser scanning path (Cheng et al., 2024). Furthermore, the surface of metallic scaffolds can be modified through techniques like sandblasting, acid etching, and anodization to enhance surface roughness and bioactivity, promoting cell adhesion and proliferation (Zhang et al., 2023e; Liu et al., 2019). However, metallic materials inherently lack bioactivity and have a significant difference in elastic modulus compared to bone tissue, which can lead to stress shielding. Future research should focus on developing novel bioactive metallic materials, optimizing surface modification methods for metallic scaffolds to enhance bioactivity and osseointegration, and investigating the *in vivo* degradation behavior and long-term biocompatibility of metallic scaffolds to achieve their clinical application in tendon-bone interface regeneration.

A comparative summary of the Performance of gradient biomimetic scaffolds currently used for the tendon-bone interface is presented in Table 5.

**TABLE 5:** Performance comparison of gradient biomimetic scaffolds.

Scaffold	Pore Size	Porosity	Water Absorption (%)	Wettability	Young’s Modulus	Degradation Rate	Cell Compatibility
Col-HAP Scaffold (Dai et al., 2024)	100–200 μm	–	523.7% ± 30.9%	HAP layer: 80.4 ± 0.3° Col layer: 89.1 ± 1.44°	23.2 ± 3.8 kPa	14days degraded by 50%	Promoted hAMSCs adhesion, osteogenesis and chondrogenesis
Gelatin-GelMA Scaffold (Zhong et al., 2025)	87.9 ± 5.6 μm	–	424.8% ± 9.9%	–	181.48 ± 29.94 kPa	25days degraded by 50%	Promoted chondrogenic or osteogenic differentiation via mechanical loading
Silk Fibroin Scaffold (Li et al., 2023)	132–255 μm	–	–	–	31.4 kPa	21dayays degraded by 17.1%	–
PCL-TCP Porous Scaffold (Cao et al., 2020)	Top layer: 78.2% ± 0.1% Bottom layer: 59.3% ± 0.4%	–	36.0% ± 3.2%	–	55 MPa	17days degraded by 17%	Cell viability on day 7: 91.4% ± 2.6% (top), 89.2% ± 3.6% (bottom)
PCL/PLA Scaffold (Baudequin et al., 2017)	–	80%–84%	–	122.7° ∼ −130°	15–60 MPa	–	Promoted cell proliferation and osteogenic differentiation after 96h
PCL-BCP Scaffold (Supphaprasitt et al., 2022)	500 μm	–	–	–	34.75 ± 3.52 MPa	90days degraded by 10.48%	Promoted cell proliferation and osteogenesis
40%MNBG-PLGA Scaffold (Tian et al., 2019)	68.74 ±19.68 μm	–	–	–	1.13 ± 0.058 MPa	28days degraded by 20%	Promoted BMSC viability, proliferation and differentiation

### 4.5 Current status of polymeric materials in tendon-bone interface regeneration based on bibliometric analysis

**FIGURE 3:**
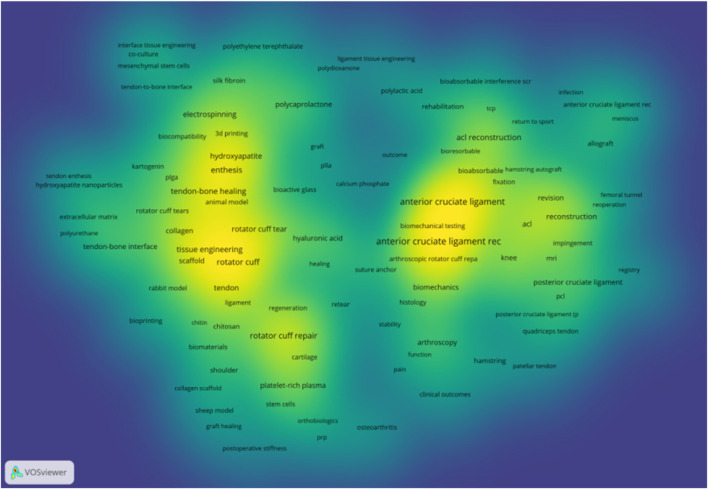
Keyword density map of polymeric materials for tendon-bone interface.

To systematically analyse the research landscape of polymeric scaffolds for TBI regeneration, we performed a bibliometric analysis using a precise keyword strategy based on the Web of Science Core Collection. The keyword density map (Figure 3) reveals two core and interconnected research clusters within the TBI repair field. The left and central areas represent the tissue engineering and regenerative medicine cluster, while the right side represents the clinical surgery cluster. These two clusters are linked by terms such as “Healing,” illustrating the research panorama from cutting-edge laboratory exploration to clinical application.

To further analyse the application of various biomaterials in TBI regeneration research, we statistically analysed the occurrence frequency and total link strength of relevant keywords within the core literature database. As shown in Table 6, among all analysed materials, hydroxyapatite exhibits the highest occurrence frequency and total link strength. This demonstrates that introducing an osteoinductive inorganic phase is an indispensable key strategy in mimicking the gradient transition from soft (tendon) to hard (bone) within the TBI. Among natural polymers, hyaluronic acid ranks first, highlighting its crucial role in maintaining tissue hydration, lubricating joints, and serving as a key component of the fibrocartilage extracellular matrix. Collagen, however, exhibits a higher total link strength than hyaluronic acid, reflecting its integral role as an organic component of the native tendon-bone interface. Among synthetic polymers, polycaprolactone (PCL) has the highest occurrence frequency. Its excellent mechanical toughness, controllable degradation profile, and suitability for 3D printing/electrospinning make it an ideal material for constructing the main scaffold structure and providing long-term mechanical support.

**TABLE 6:** Keyword occurrence frequency and total link strength of polymeric materials for tendon-bone interface.

Tape	name	occurrences	total link strength
Natural polymers	hyaluronic acid	15	27
	collagen	13	32
	silk fibroin	10	20
	chitosan	9	22
	alginate	3	8
Synthetic polymers	polycaprolactone	15	41
	polylactic acid	6	13
	plga	6	17
	polyurethane	3	5
Inorganic biomaterials	hydroxyapatite	19	50
	bioactive glass	4	9
	tcp	3	5

## 5 Fabrication techniques for gradient biomimetic scaffolds

Gradient biomimetic scaffolds aim to mimic the complex, continuously varying microenvironmental characteristics present in native tissues, such as spatial distribution gradients of extracellular matrix components, structure, mechanical properties, and bioactive factors. These gradients are crucial for guiding cell behavior and regulating tissue development and regeneration (Pattnaik et al., 2023). Therefore, developing fabrication techniques that can precisely construct scaffolds with specific gradient features has become a research hotspot in the field of tissue engineering. In recent years, various advanced manufacturing techniques have been applied to the construction of gradient scaffolds, mainly including electrospinning, additive manufacturing (3D printing), phase separation/freeze-drying, and some emerging techniques such as microfluidics and modular assembly.

### 5.1 Electrospinning and gradient construction methods

Electrospinning is a key nanofiber fabrication technique that utilizes a high-voltage electrostatic field to stretch a polymer solution or melt through a needle tip, forming nano/micro-scale fibers that are ultimately deposited on a collector to form a nonwoven fiber mat (George and Luo, 2020). Due to its ability to fabricate scaffolds that mimic the fibrous structure of the native extracellular matrix and its advantages of high porosity and large specific surface area (Ding et al., 2021), electrospinning technology exhibits great application potential in tendon-bone interface repair. To mimic the complex biological and physicochemical gradients of the TBI region from tendon to bone, researchers have developed various electrospinning-based gradient scaffold construction strategies.

Layer-by-Layer Electrospinning: Layer-by-layer electrospinning is one of the most direct methods for achieving compositional or structural gradients in biomimetic scaffolds. By electrospinning polymer solutions with different compositions, concentrations, or physical properties in a predetermined sequence, fiber scaffolds with layered gradient structures can be formed (Luo et al., 2021). For example, Shang et al. constructed a biomimetic gradient sandwich nanofiber scaffold with mechanical properties and biocompatibility by layer-by-layer electrospinning of silk fibroin composites. This scaffold promoted the adhesion, proliferation, and infiltration of osteoblasts and effectively regulated the inflammatory response, successfully promoting functional tissue regeneration at the tendon-bone interface (Shang et al., 2025). However, this method may lead to insufficient interlayer bonding, forming distinct interfaces rather than a smooth, continuous gradient (Bayrak et al., 2017).

Coaxial Electrospinning: This technique can fabricate single fibers with core-shell or more complex multilayer structures (Ghasemkhah et al., 2019). By encapsulating bioactive molecules such as growth factors in the shell or core layer and controlling their concentration or polymer degradation rate, gradient release of active factors can be achieved (Ghasemkhah et al., 2019). One study used coaxial electrospinning to encapsulate VEGF in a polymer and successfully controlled the gradient release rate of VEGF by changing the shell thickness or polymer type, aiming to promote a vascularization gradient (Liu et al., 2023).

Emulsion Electrospinning: As one of the modified electrospinning techniques alongside coaxial and blend electrospinning, emulsion electrospinning can efficiently load various bioactive substances or nanoparticles into fibers and can provide the possibility for constructing gradient scaffolds by building core-shell structures (Norouzi et al., 2015). For example, one study used emulsion electrospinning to prepare a gradient fiber scaffold rich in platelet lysate and nano-hydroxyapatite. This scaffold was able to continuously release bioactive factors, promoting region-specific differentiation of tenocytes and osteoblasts, thereby optimizing the TBI repair effect (Calejo et al., 2022).

Despite the many advantages and flexibility that electrospinning exhibits in constructing TBI gradient scaffolds, it still faces some challenges. First, the average pore size of electrospun scaffolds is usually small (often less than 10 μm) (Matsuzaki et al., 2020), which may hinder the effective penetration of cells into the scaffold interior, affecting the regeneration of deep tissues. Second, the organic solvents commonly used in electrospinning, such as dichloromethane, trifluoroethanol, and hexafluoroisopropanol, may have toxic effects on cells due to their residues, affecting the biocompatibility of the scaffold and limiting its clinical translation (Jiang et al., 2019). Furthermore, electrospinning struggles to replicate the mineral gradient of native tendon-bone tissue along the fibre alignment direction, which can affect the precise mechanical integration and signal transduction between the scaffold and the regenerating tissue (Chen Y. et al., 2024). Finally, the electrospinning process is complexly influenced by various factors such as electric field strength, solution flow rate, and environmental humidity, which poses challenges to achieving fiber uniformity and stability in large-scale production. Overcoming these limitations is a key focus of future research to fully realize the potential of electrospinning technology in the field of TBI regeneration and repair.

### 5.2 Application of additive manufacturing techniques in constructing gradient biomimetic scaffolds for tendon-bone healing

Additive manufacturing (AM) techniques, commonly known as 3D printing, are a novel manufacturing approach based on computer-aided design models that builds three-dimensional objects by precisely accumulating materials layer by layer. AM has demonstrated tremendous potential in tissue engineering and regenerative medicine, particularly in the repair of the complex tendon-bone interface, where it has become a research hotspot (Kim et al., 2025). The core advantages of this technology lie in its high precision, customizability, and the ability to freely and controllably construct complex structures such as the internal microstructure and overall morphology of scaffolds, making it well-suited for fabricating biomimetic scaffolds that mimic the complex gradients of the native TBI (Cao et al., 2020). Various additive manufacturing techniques, such as fused deposition modeling, stereolithography, and selective laser sintering, have been successfully applied to the fabrication of gradient scaffolds.

Controllable Fabrication of Material Composition Gradients: Using multi-nozzle or hybrid printing head systems, additive manufacturing techniques can precisely control the ratio and spatial distribution of different bioinks containing different polymers, inorganic particles, and bioactive molecules during the printing process, thereby constructing scaffolds with continuous composition gradients (Sinha et al., 2021). For example, researchers used extrusion-based bioprinting technology to successfully prepare hydrogel scaffolds with continuous mechanical property gradients by dynamically changing the mixing ratio of two hydrogels with different stiffnesses, mimicking the mechanical environment gradient of soft tissues (Kuzucu et al., 2021). Similarly, by printing bioinks containing different concentrations of bioactive factors or different types of cells, biochemical gradients or cell density gradients can be directly constructed within the scaffold to guide region-specific cell behavior (Zhang et al., 2020).

Optimization of Mechanical Property Gradient Construction: Additive manufacturing techniques allow for the integration of different materials by rationally adjusting printing parameters such as exposure time, light intensity, printing infill, laser pumping current, and printing speed, or by introducing reinforcing phases (Traxel and Bandyopadhyay, 2020), thereby optimizing the strength and stiffness of the scaffold so that it exhibits gradient mechanical properties in different regions that conform to the physiological characteristics of the TBI. One study designed a 3D-printed scaffold enhanced with hydroxyapatite and multifunctional nanoparticles. By adjusting the printing parameters, the modulus of the scaffold reached 143.8 GPa and the porosity reached 59.6% (Singh I. et al., 2023), achieving significant optimization of mechanical properties and demonstrating the potential of 3D printing for controlling mechanical gradients.

Multi-Gradient Integration Capability: One of the core advantages of additive manufacturing technology is its ability to integrate multiple gradients (such as structural gradients, composition gradients, mechanical gradients, and bioactivity gradients) into the same scaffold, thereby achieving a deeper and more comprehensive simulation of native tissues. For example, complex scaffolds with both mechanical gradients and pore structure gradients can be constructed by controlling printing structural parameters to more accurately mimic the heterogeneity of native tissues (Elenskaya et al., 2023; Günther et al., 2022). Extrusion-based bioprinting technology can even directly distribute different types of cells in a gradient pattern within the scaffold during the printing process, achieving integrated gradient construction of structure, composition, and bioactivity (Kuzucu et al., 2021).

Despite the significant advantages of additive manufacturing techniques in constructing TBI gradient scaffolds, some challenges remain. The rheological properties of bioinks can affect the accuracy and stability of printing. Some printing materials may cause structural collapse during printing or subsequent applications due to their insufficient mechanical properties, affecting the mechanical stability of the scaffold. Meanwhile, 3D printing technology faces challenges in printing fibres with diameters that match the physiological scale (e.g., the nanoscale of collagen fibrils), impacting cell adhesion, proliferation, and migration (Yang et al., 2022). In addition, the printing speed of additive manufacturing techniques is relatively slow, especially for printing high-precision and complex structures, which may limit their large-scale production and clinical translation efficiency. The traditional single-layer step-by-step stacking printing method often reduces production efficiency while improving accuracy, and the printing process of complex structures may require lengthy process optimization and post-processing. Overcoming these technical bottlenecks will be key to promoting the widespread application of 3D-printed gradient scaffolds in the field of TBI repair.

### 5.3 Other emerging techniques

In addition to the mainstream techniques mentioned above, some emerging strategies are also demonstrating unique potential in gradient scaffold construction.

Microfluidics: Microfluidic technology enables precise manipulation of fluids at the micron scale. By designing specific microchannel networks and flow fields, concentration gradients and other features can be generated (Perrodin et al., 2020). Microfluidic technology can be used to fabricate hydrogel microenvironments with precise chemical or bioactive factor gradients (Santo et al., 2016). Furthermore, microfluidic emulsion methods can be used for high-throughput preparation of monodisperse microspheres, which can then be used as cell carriers in tissue engineering. For example, Wu et al. developed a high-efficiency microfluidic emulsion system using an integrated microchannel plate (MCP), achieving high-throughput preparation of monodisperse droplets and demonstrating that the microspheres prepared with this system have good cell compatibility, providing a new platform for gradient scaffold applications (Wu et al., 2023). By forming a growth factor concentration gradient in a microfluidic chip based on the principle of diffusion and then performing *in situ* photopolymerization, hydrogel scaffolds embedding a growth factor gradient signal can be fabricated (Santo et al., 2016).

Modular Assembly: This strategy involves assembling pre-fabricated micro-units with different characteristics (such as microgels, cell spheroids, or micro-scaffold modules) according to a pre-determined design to form a macroscopic gradient structure (Caldwell et al., 2020). This “bottom-up” construction method is highly flexible and easy to integrate with multiple functional gradients. For example, microgel modules containing different cell types or different ECM components can be arranged in a gradient and fused together to construct complex gradient structures that mimic tissue interfaces (Jalandhra et al., 2023).

The fabrication of gradient biomimetic scaffolds is one of the key challenges facing the field of tissue engineering. Electrospinning, additive manufacturing, and other techniques provide diverse strategies for constructing scaffolds with different types of gradients. Electrospinning excels at fabricating fibrous gradient structures, additive manufacturing has clear advantages in the precise control of macroscopic and microscopic structures, and emerging techniques such as microfluidics and modular assembly provide new ideas and tools for gradient construction. In the future, combining the advantages of different techniques to develop multi-scale, multi-functionally integrated gradient scaffold fabrication methods will be an important direction for promoting tissue regeneration research and clinical applications. The choice of which technique to use depends on the specific characteristics of the target tissue, the type and precision of the required gradients, and the needs of the actual application.

A comparative summary of the manufacturing techniques for the aforementioned three types of gradient biomimetic scaffolds is presented in Table 7.

**TABLE 7:** Manufacturing technologies of gradient biomimetic scaffolds.

Manufacturing Technology	Method	Advantages	Limitations	Applicability for Constructing Gradient Scaffolds
Electrospinning	Layer-by-layer Electrospinning (Shang et al., 2025)	Simple and intuitive; suitable for component/structural layering; builds mechanical and compositional gradients	Weak interlayer bonding; clear interfaces may form; lack of continuous gradient	Layer-by-layer stacking enables compositional and structural differences, but the gradient is discontinuous and interlayer adhesion is weak. Suitable for simple stratified biomimetic structures
	Coaxial Electrospinning (Liu et al., 2023)	Enables core-shell structure and controlled gradient release of growth factors	Complex process; high parameter control requirements	Enables precise core-shell structure construction and gradient release of bioactive factors; ideal for microscale functional gradients, though overall structural control is limited
	Emulsion Electrospinning (Calejo et al., 2022)	Efficiently loads various bioactive molecules and nanoparticles; builds core-shell structures	Small pore size, poor cell infiltration; residual solvents may be cytotoxic	Suitable for embedding multiple components and fabricating bioactive composite fibers; simple to form, but slightly less precise than coaxial electrospinning
Additive Manufacturing (3D Printing)	Composition Gradient Printing (Kuzucu et al., 2021; Zhang et al., 2020)	Multi-nozzle/mixed-nozzle systems control ratio of bioinks to build compositional and biochemical gradients	Rheological properties of bioinks affect print precision; some materials have low strength	Allows precise control of material and factor distribution via multi-nozzle mixing, enabling continuous compositional and cellular gradients; ideal for multi-tissue interfaces
	Mechanical Gradient Optimization (Singh et al., 2023b)	Adjusts print parameters to control material stiffness and porosity	Slow printing speed; complex structure requires long post-processing	Enables tuning of material stiffness and porosity to achieve a continuous mechanical gradient from soft to stiff; well-suited for mimicking the physiological mechanics of the tendon–bone interface (TBI)
	Multiple Gradient Integration (Kuzucu et al., 2021; Elenskaya et al., 2023; Günther et al., 2022)	Simultaneously constructs gradient of structure, composition, cell density, and bioactivity	Difficult to print physiological fiber diameters; limited industrial scalability	Integrates all gradient construction capabilities; ideal for fabricating highly biomimetic and heterogeneous tissue scaffolds. Currently the most promising integrative approach
Emerging Technologies	Microfluidics (Santo et al., 2016; Wu et al., 2023)	Precisely generates chemical/growth factor gradients; ideal for microscale environments	Mainly limited to microscale structures; challenges in macro-level integration	Offers precise control of chemical and growth factor gradients at the microscale, but not suitable for large-scale structural or mechanical construction. Best used to regulate localized signaling microenvironments
	Modular Assembly (Jalandhra et al., 2023)	Uses functional micro-units (e.g., microgels, spheroids) to form macroscale gradient structures; bottom-up strategy	Challenges in stable module integration and whole-structure gradient control	Flexible assembly of functional modules enables diverse gradient combinations and zonal cellular arrangement; however, overall macrostructural stability remains a challenge

## 6 Summary and outlook

Gradient biomimetic scaffolds, as an important strategy in the field of tissue engineering for mimicking the complexity of native tissues, have achieved significant progress in recent years. They have demonstrated tremendous potential, especially in treating tissues with complex interface structures such as tendon-bone, ligament-bone, or osteochondral tissues, laying the foundation for achieving functional reconstruction of interfaces like the tendon-bone junction.

The core advantage of gradient biomimetic scaffolds lies in their high degree of mimicry of the native tissue microenvironment. By constructing continuous variations in physical (e.g., porosity, stiffness gradient), chemical (e.g., material composition, surface chemical modification gradient), or biological (e.g., growth factor, drug concentration gradient) parameters within the scaffold, cell behavior such as migration, adhesion, differentiation, and angiogenesis can be guided more precisely. Studies have shown that physical gradients can effectively guide lineage-specific differentiation of cells, while biological gradients can mimic the spatiotemporal distribution of signaling molecules *in vivo*, enabling precise spatiotemporal control of cell behavior. Currently, various techniques are used to fabricate gradient scaffolds, including electrospinning (achieving gradients in fiber diameter, orientation, porosity, and molecular concentration), additive manufacturing (particularly multi-material printing, achieving precise three-dimensional structures and complex composition gradients), microfluidics (precisely controlling concentration gradients), and modular assembly. Overall, gradient scaffolds enhance the efficiency and functionality of tissue regeneration by providing complex cues that are closer to the physiological state.

Currently, the translation of gradient biomimetic scaffolds for tendon-bone healing from laboratory research to clinical application still faces many challenges. ① Precision and Hierarchy of Structural Biomimicry: Most current scaffolds are still based on “macroscopic layered structures” (such as three-layer designs), making it difficult to truly mimic the continuous gradient characteristics of native interfaces (such as the tendon-bone interface) at multiple scales, including microstructure arrangement, collagen fiber orientation, and angle. Precisely controlling the gradient profile at the nanoscale, achieving synergistic construction of complex gradients with multiple parameters (e.g., structure-composition-cell), and ensuring high batch-to-batch reproducibility remain significant challenges. ② Spatiotemporal Control and Stability of Biological Function Gradients: Existing scaffolds are mostly “static gradients,” making it difficult to dynamically adapt to the needs of each stage of tissue regeneration. Chemical and biological gradients may gradually disappear or deform *in vivo* due to material degradation and molecular release/inactivation, making it difficult to maintain their long-term stability and gradient effectiveness. In addition, when relying on growth factors or co-culture to construct cell lineage gradients, cell migration can easily lead to blurred interfaces and decreased gradient stability. ③ Vascularization: For large-volume defects, rapid and effective vascularization is a key bottleneck. Although gradients can be designed to guide angiogenesis, how to form a functional and stable capillary network throughout the scaffold (especially in the central region) and successfully anastomose it with the host vascular system remains extremely challenging. The gradient structure itself may also affect the uniform distribution of the vascular network. ④ Immunomodulation: Many biomaterials, upon implantation, can trigger varying degrees of local or systemic inflammatory responses, potentially leading to the formation of foreign body giant cells and fibrous encapsulation, which in turn impair scaffold integration and tissue regeneration. How to design gradient scaffolds to actively regulate the local immune microenvironment, guide polarization towards a reparative M2 phenotype, and avoid chronic inflammation and fibrous encapsulation is key to achieving long-term functional regeneration.

To address these challenges and promote clinical translation, future development should focus on: ① Intelligence and Dynamism: Developing “smart” or “4D” gradient scaffolds that can respond to endogenous signals (pH, enzymes) or external stimuli (light, magnetism, ultrasound) to achieve dynamic changes and on-demand regulation of gradient characteristics, better mimicking the time-varying microenvironment and achieving precise intervention. ② Multi-Functional Integration and Multi-Gradient Synergy: Integrating multiple types of gradients (physical, chemical, biological) into a single scaffold to more comprehensively mimic the complex tissue microenvironment. Exploring hybrid processes that combine different technologies (e.g., “additive manufacturing + microfluidics + micro/nano deposition”) to form a comprehensive manufacturing system with spatial precision, local function, and overall structural synergy. At the same time, integrating sensing or imaging elements to achieve non-invasive monitoring of the regeneration process. ③ New Strategies for Cell Gradient Construction: Strengthening research on platforms that induce differentiation of single stem cell lineages based on physical cues (stiffness gradient, nanotopography) or external field (electric/magnetic) responses, and developing “boundary-free induction-type” cell gradient construction strategies to improve biosafety and operational simplicity. ④ Personalized Customization: Combining medical imaging and high-resolution additive manufacturing to design and manufacture personalized gradient scaffolds based on the patient’s specific condition. ⑤ Clinical Translation-Oriented Research: Strengthening preclinical validation in large animal models, conducting long-term functional assessments under load, and establishing a comprehensive evaluation system. Focusing on the standardization, quality control, scalable production processes, surgical operability, and host integration of scaffolds.

The current commercialisation status of graded biomimetic scaffolds also warrants attention. While promising progress has been made in the laboratory setting, there are currently no commercially available biomimetic scaffolds that replicate the continuous gradient of the native tendon-bone interface and have received regulatory approval for market release. This is primarily due to several bottlenecks in translating biomimetic scaffolds from the laboratory to commercial production: First, there are challenges related to manufacturing complexity and cost control. The intricate gradient structures demand stringent requirements for materials, process stability, and quality control, leading to high production costs and difficulties in achieving cost-effective large-scale manufacturing. Second, the regulatory approval pathway is lengthy and rigorous. These highly complex scaffolds, often containing bioactive molecules, are typically classified as Class III medical devices. Their safety and efficacy must be demonstrated through extensive and expensive preclinical (especially large animal models) and clinical trials, resulting in long approval timelines and substantial investment. Third, market access and clinical acceptance are crucial. New products must demonstrate a significantly improved cost-benefit ratio compared to existing treatment options to persuade clinicians, hospitals, and healthcare payers to adopt them, despite potentially higher upfront costs. Despite these challenges, graded biomimetic scaffolds hold significant commercial promise. The suboptimal outcomes of traditional tendon-bone injury repair techniques leave a substantial market opportunity for products that genuinely promote tendon-bone interface regeneration. Furthermore, advancements in additive manufacturing (3D printing), AI-assisted process optimisation, and the development of novel smart bioinks are expected to significantly reduce production costs, improve process stability, and address the challenges of large-scale manufacturing in the future. More importantly, graded biomimetic scaffolds represent a paradigm shift in treatment philosophy from “mechanical suturing” to “biological functional reconstruction.” Successful translation will not only improve surgical success rates but also enhance patients’ long-term quality of life and reduce overall healthcare costs, including those associated with revision surgeries and rehabilitation. Therefore, promoting the market entry of biomimetic scaffolds requires not only continuous innovation in basic research but also close collaboration between industry, academia, research institutions, and clinicians to overcome key challenges in standardised production, long-term efficacy validation, cost control, and regulatory strategies.

## 7 Conclusion

Regenerating the complex tendon-bone interface (TBI) remains a significant challenge. Gradient biomimetic scaffolds offer a sophisticated strategy, mimicking the TBI’s native structural and biological gradients to guide organized tissue formation. While advanced fabrication techniques show promise in constructing these architectures using diverse biomaterials, critical hurdles persist. Achieving high-fidelity biomimicry, ensuring long-term stability, and promoting vascularization are paramount. Future innovations in smart materials and focused translational studies are essential to unlock the full clinical potential of these scaffolds for robust and functional TBI regeneration, moving beyond preclinical successes towards tangible patient benefits.
